# Gum Arabic as fetal hemoglobin inducing agent in sickle cell anemia; in vivo study

**DOI:** 10.1186/s12878-015-0040-6

**Published:** 2015-12-29

**Authors:** Lamis Kaddam, Imad FdleAlmula, Omer Ali Eisawi, Haydar Awad Abdelrazig, Mustafa Elnimeiri, Florian Lang, Amal M. Saeed

**Affiliations:** Department of Physiology Faculty of Medicine, Alneelain University, P.O. Box: 11121, Khartoum, 12702 Sudan; Alneelain Research Centre Faculty of Medicine, Alneelain University, Khartoum, Sudan; Department of Hematology, Military Hospital, Khartoum, Sudan; Department of Pediatrics, Military Hospital, Khartoum, Sudan; Department of Community Medicine Faculty of Medicine, Alneelain University, Khartoum, Sudan; Department of Physiology, University of Tübingen, Tübingen, Germany; Department of Physiology Faculty of Medicine, University of Khartoum, Khartoum, Sudan

## Abstract

**Background:**

High levels of fetal haemoglobin (HbF) decrease sickle cell anaemia (SCA) severity and leads to improved survival. According to in vivo and in vitro studies, butyrate increases HbF production. Its utilization in clinical practice is hampered, however, by its short half-life. Serum butyrate concentrations could be enhanced by colonic bacterial fermentation of Gum Arabic (GA), edible, dried, gummy exudates from Acacia Senegal tree. We hypothesized that regular intake of GA increases serum butyrate levels, thus inducing HbF production and ameliorating symptoms of sickle cell anemia.

**Methods:**

Fourty seven patients (5–42 years) carrying hemoglobin SS were recruited from April 2014 to January 2015. Patients received 30 g/day GA for 12 weeks. HbF, blood count and erythropoietin level were measured. The main outcome of interest was the level of HbF after 12 weeks. The secondary outcomes were improvement in clinical and laboratory results. The study was ethically approved by Alneelain University IRB.

**Results:**

The study revealed significant increase in HbF level P.V0.000 [95 % CI, 0.43–1.02], MCV P.V:000 [95 % CI, 2.312–6.058] and Hematocrit level P.V:0.026 [95 % CI, 0.124–1.902]. No significant difference was encountered in platelets count P.V: 0.346 [95 % CI,−25.76–71.94], and WBCs count P.V:0.194 [95 % CI,−8.035–1.68]. Thirty seven percent of patients experienced minor side effects which resolved within a week.

**Conclusion:**

These findings reveal a novel effect of GA, which may be used to foster fetal hemoglobin production.

**Trial registration:**

ClinicalTrials.gov Identifier: NCT02467257. Registered 3rd June 2015.

**Electronic supplementary material:**

The online version of this article (doi:10.1186/s12878-015-0040-6) contains supplementary material, which is available to authorized users.

## Background

Homozygous sickle cell Anemia (SCA) is an autosomal recessive genetic disease that results from the substitution of valine for glutamic acid at position 6 of the β-globin chain, leading to production of hemoglobin S (HbS) [1]. HbS polymerizes in red blood cells upon deoxygenation. This causes the RBCs to change from biconcave disc shape to an irregular sickled shaped. Sickled RBCs can block blood vessels, and thus decrease the delivery of oxygen to organs and tissues. Sickled cells are extremely susceptible to hemolysis and eryptosis [2], causing chronic anemia [3]. Sickle cell disease (SCD) is the most common genetic disorder among people of African descent [4].

Fetal hemoglobin (HbF) expression is a crucial determinant of the clinical severity of SCD [5]. The percentage of HbF (HbF%) influences both laboratory values and clinical features of children and adults with sickle cell anemia [6]. These observations were largely responsible for the shift of therapeutic emphasis and strategies to increase the level of HbF in vivo in patients with sickle cell disease [1]. Hydroxyurea (HU) been approved by the FDA to treat adult sickle cell patients [7]. Still HU is underutilized because of concern regarding safety and lack of availability in many parts of the developing world [8]. In addition HU is expensive [9] and requires regular follow up to assess response and monitor toxicity, which restrict it is usage even more in low resources setting. Both in vivo and in vitro studies demonstrate that butyrate administration similarly increases Hemoglobin F production [10–14]. So far the chemical derivatives of butyrate are of less clinical value because of their low bioavailability and rapid metabolism [8]. Arginine butyrate had to be given by continuous intravenous infusion in large volumes, and sodium phenylbutyrate required as many as 40 tablets daily [8]. Butyrate could, however, be generated from Gum Arabic (GA), edible, dried, gummy exudates from the stems and branches of Acacia Senegal and Acacia Seyal, rich in non-viscous soluble fiber. It is defined by the FAO/WHO Joint Expert Committee for Food Additives (JECFA) as a dried exudation obtained from the stem of A. Senegal [15]. GA has wide industrial uses as a stabilizer, thickening agent and emulsifier, mainly in the food industry (e.g. in soft drinks syrup, gummy candies and marshmallows). The US FDA recognized it as one of the safest dietary fibres [15, 16]. GA is indigestible for both human and animals; Its fermentation by colonic intestinal bacteria leads to formation of various degradation products, such as short-chain fatty acids [17]. Gum Arabic ingestion increases serum short chain fatty acid concentration, mainly butyrate and propionate [15, 18]. Serum butyrate concentration increased following administration of GA in healthy subjects [15, 19]. Oral intake of GA has been shown to provide several health benefits [20], such as prebiotic effects [16]. GA significantly increases Bifidobacteria, Lactobacteria, and Bacteriodes in the gut [16]. GA is claimed to have anti-cancer [16], anti-malarial [17] immune-modulatory [17, 21] and antioxidant effects [15, 16, 22]. GA treatment has been shown to favorably influence clinical and laboratory results in rats with adenine-induced chronic renal failure CRF and in humans diagnosed with renal failure [15, 17, 21]. GA shown to increase Erythropoietin level In two separate studies and ameliorated anemia caused by adenine administration [23, 24].

We hypothesized GA degradation delivers short chain fatty acids, which in turn have been shown to stimulate fetal hemoglobin expression in RBCs. Increased levels of erythrocyte fetal hemoglobin are known to hinder the intraerythrocytic HbS polymerization and provide some protection against hemolysis and vaso-occlusive crisis [5]. The present study tested whether Gum Arabic may influence the clinical course of SCD.

To the best of our knowledge this is the first study conducted to investigate the effect of oral administration of GA on fetal hemoglobin production in sickle cell anemia patients.

## Methods

This is an experimental study with the aim to produce primary data for hematological efficacy of oral intake of Gum Arabic as fetal hemoglobin inducer in sickle cell anemia patients. The participants were recruited from the out patients clinic of pediatric and adult hematology units in Military hospital-Khartoum-Sudan. Inclusion criteria were: patients homozygous for SCD (SS) as documented by Hemoglobin electrophoreses, aged between 5 and 50 years. The total number of participants recruited mounted to 47. All medications and dosages had been stable for 2 weeks before study entry. All participants received folic acid 5 mg daily to support erythropoiesis. Exclusion criteria: patients received blood transfusion within the last 3 months or admitted to the hospital within 2 weeks because of SCD-related events or crisis. Ethical clearance was obtained from the Institutional Review Board at Alneelain University and from Research Ethics Committee- Khartoum State Ministry of Health. Principal investigator obtained informed consent from each participant or from parents when the patient is less than 18 years old prior to the interview.

### Gum arabic administration

GA in powder form, it is a 100 % natural extract powder produced mechanically from the wildly grown Acacia Senegal tree with a particle size less than 210 μm. GA in powder form was provided from Dar Savanna Ltd., Khartoum, Sudan. Properties and composition of GA are listed elsewhere [22]. The daily dose was 30 gram. The dose was determined based on previous studies [16, 19]. It was given in one sachet to be consumed early morning dissolved in water for 12 weeks. The GA was provided to the participants every 2 weeks for 3 months (14 sachets per each visit). Empty sachets were retained every visit as indicator of compliance.

A pre-coded and pre-tested standardized questionnaire and check list were used to collect data about participants’ physical examination, weight, height, severity of the symptoms and any side effects. Clinical safety assessments and laboratory tests, complete blood count (CBC) every 2 weeks using automated cell counter (Sysmex) were regularly conducted. In addition serum chemistry including renal function tests (RFT) and Liver function test (LFT) were carried out every 4 weeks. Regular follow up was carried by the consultant physician in the unit.

Hemoglobin F was measured by modified fully automated capillary2 flexpiercing hemoglobin electrophoresis technique (Sepia France) prior to starting GA and then every 4 weeks. Plasma was separated from EDTA sample and used for measurement of Erythropoietin (EPO) level by enzyme-linked immunosorbent assay (ELISA) using “Wkea, USA” EPO kit.

Data were analyzed using SPSS version 20. Paired samples *T* test was used to compare between pre and post intervention results. P values equal or less than 0.05 was considered significant.

## Results

A total of 47 patients were enrolled (Table 1) between April, 2014 (first subject enrolled) to January, 2015 (last patient last visit) when adequate data were collected to allow for planning for further study. All were Sudanese; 23 were males (age 5 to 42 years). Seven patients were on a stable dose of hydroxyurea 500gram per day.

**Table 1:** Demographics and baseline characteristics

Characteristics	Mean	SD	Median	Maximum	Minimum
Age	16.26	8.52	15	42	5
Gender	23(49 %) Male				
Base line weight (Kg)	35 · 96	14	37 · 3	63	13
Base line height (Cm)	148 · 34	20.99	154 · 5	107	190
Hb g/dL	7.28	1.105	7	11	5 · 5
Hb F (%)	6.68	5.44	4.80	17.50	00
Hb S (%)	89 · 99	5 · 15	91	97 · 20	79 · 40
Hb A_2_(%)	3 · 33	0 · 52	3 · 3	4 · 4	2 · 5

Duration of treatment was for 12 weeks except two patients received GA for 9 weeks and eight patients for 10 weeks. The last recorded results were considered for final analysis as post treatment results. Four patients were excluded because of blood transfusion during first 2 weeks of the study. One patient taking GA for 10 weeks was excluded because he developed severe malaria requiring blood transfusion.

**Fig. 1:**
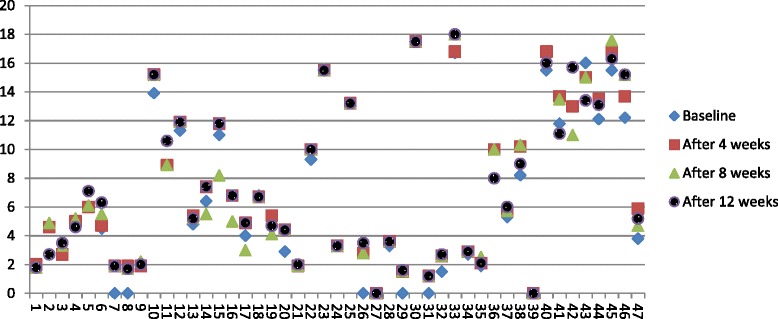
Hb F value from baseline to week 12, by subject. Each symbol represents one patient. Patients 41–47 are on HU dose

Daily oral intake of GA significantly increased HbF level, MCV and hematocrit (Table 2). Peak HbF was recorded after 4 weeks and sustained till week 12 for most of the patients (Fig. 1 and Additional file 1). GA treatment was not followed by significant increase in hemoglobin concentration or MCH (Table 2). There was no significant change in WBC counts or Reticulocyte count (Table 2) and no significant increase in Erythropoietin level. A positive correlation was observed between absolute change in HbF (∆F) level and Erythropoietin level (∆Epo) (Pearson Correlation. 383, P. value 0 · 04 95 % CI 0 · 019to 0 · 66). Thirteen patients (28 %) have been admitted once to hospital while two patients were admitted twice. All were admitted for 24 h because of painful crisis. One patient has chronic leg ulcer healed after taking GA. There was a significant increase from base line weight by mean of 1.87Kg (P.V:0 · .0001 95 % CI 1 · 24 to 2 · 49)

**Table 2:** Comparison between the mean of pre and post intervention values of biomarkers

Variable	Base line value Mean ± SD	Post intervention concentration value Mean ± SD	P.V.	95 % CI
Hb F (%)	6·68 ± 5.44	7·41 ± 5·38	.000 ^b^	0·431–1·028
Hb S (%)	90 ± 5·15	89·24 ± 5·10	.000 ^b^	0.455–1·043
Hb A_2_ (%)	3·3 ± 0·52	3·33 ± 0.48	0·901	0·064–0·072
Hemoglobin (g/dL)	7·28 ± 1·105	7·2638 ± 1·08	0·777	−0·142–0·188
MCV (fL)	85·2 ± 9.37	89·20 ± 12·33	·000 ^b^	−2·312– − 6·058
PCV %	20·56 ± 3·15	21·57 ± 4·29	·026 ^a^	0·124–1·902
MCH pg	30·32 ± 3·87	30·01 ± 4·11	0·270	−.2496–0·871
MCHC (g/dL)	35·2 ± 2·22	33·4 ± 2·29	·000 ^b^	1·035–2·559
Reticulocyte count %	14·412 ± 4·27	16·27 ± 7·03	·185	−4·855–1·130
Platelets counts 10^3^ /uL	448·27 ± 144·79	471·36 ± 169·41	·346	−71·94–25·76
WBCs 10^3^ /uL	16·72 ± 16·3	13·54 ± 4·68	·195	−1·682–8·035
RBCs 10^6^ /uL	2·37 ± 041	2·42 ± 0·45	·170	−0·1091–0·197
LDH U/L	717·23 ± 269·95	643·14 ± 244·5	·028 ^a^	8·22–139·94
Erythropoietin IU/L	8·74 ± 2·96	8·782 ± 3·92	·926	−·944–·8617

^a^Difference is significant at the 0.05 level (2-tailed)

^b^Difference is significant at the 0.01 level (2-tailed)

### GA tolerance and side effects

37 % of patients complained from side effects such as bloating, diarrhea, nausea, and vomiting (Table 3). All these symptoms resolved spontaneously within the first 5 days. The mother of a female 5 year old patient reported that GA treatment of the child was followed by appearance of loose stool; a condition did not require intervention.

**Table 3:** Side effects of intervention among study group

Complain	Yes		NO		Total
	N	%	N	%	
Bloating	7	15	40	85	47
Diarrhea	9	19	38	81	47
Vomiting	7	15	40	85	47
Nausea	3	6	44	94	47

## Discussion

Sickle Cell Disease is the most common hemoglobin defect around the globe, with a high incidence in sub-Saharan Africa [9]. This necessitates the search for non-toxic oral and cheap therapeutic agents that increase fetal globin expression, and are tolerable for patients with minimal side effects.

**Fig. 2:**
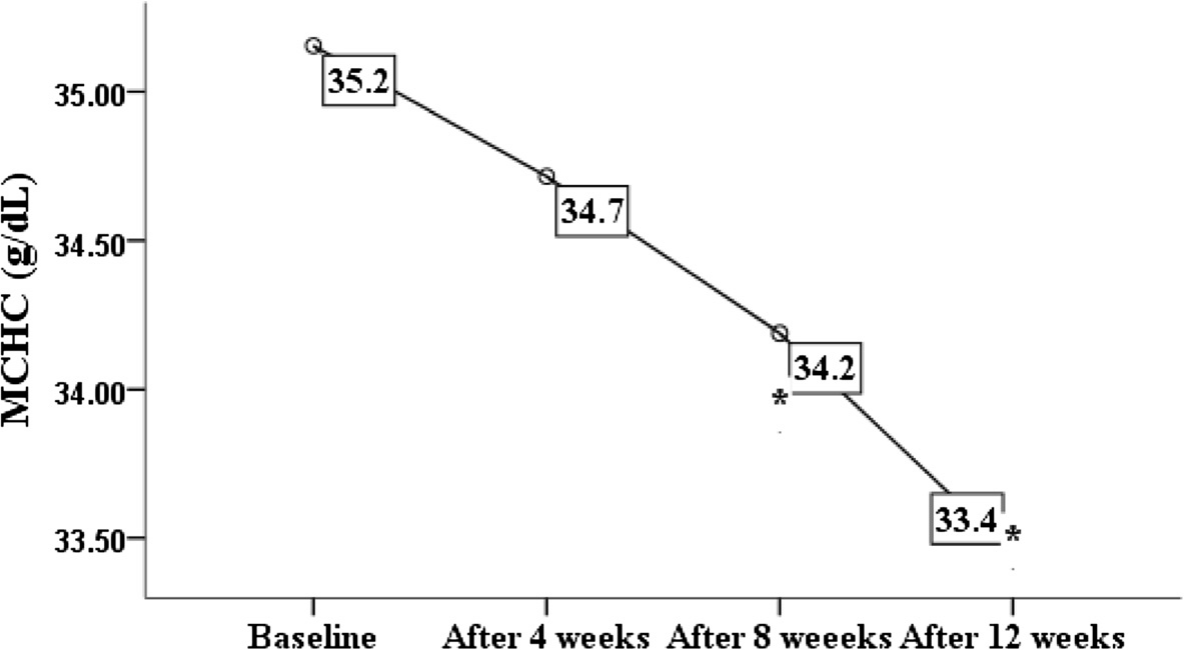
Effect of GA intake on MCV (*P* = 0.000). * indicates significant difference from baseline

According to the present study GA increases the level of HbF and significantly decreases level of HbS (Table 2). Since HbS polymerization depends on its intracellular concentration, a slight reduction is likely to have a beneficial effect on the kinetic of polymerization [25]. GA has no effect on HbA_**2**_ (P.V: 0.9), and this is expected,in spite delta chain is located in chromosome 11 like beta and gamma chains [26]. Butyrate exposure results in true reversal of switch from beta to gamma globin expression [12]. Exposure to GA increases MCV (Table 2, Fig. 2) mimicking the effect of hydroxyurea therapy [4, 6, 27–31]. Increase in MCV is linked to the increase in intracellular HbF [32] and an increase in hemoglobin F is always associated with a concomitant increase in MCV [27, 32].

**Fig. 3:**
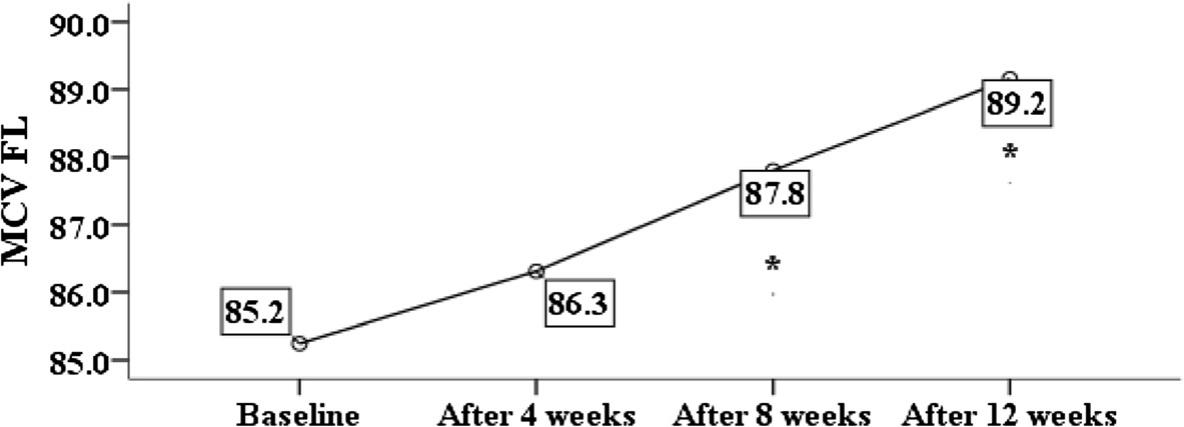
Effect of GA intake on MCHC (*P* = 0.000). * indicates significant difference from baseline

GA significantly decreased the MCHC value (Table 2 Fig. 3), an effect again mimicking effects of HU [27, 30]. Reduction in MCHC is beneficial in SCA patients since it inhibits hemoglobin S polymerization [3, 26]. GA intake did not increase hemoglobin concentration (Table 2). Total hemoglobin is increased in clinical studies following administration of parental drugs such as: 5-azacytidine, decitabine, and butyrate given for longer periods, since they allowed HbF cells to accumulate and survive [33]. Sickle cell patients have elevated lactate dehydrogenase enzyme (LDH) levels, a biological marker reflecting intravascular hemolysis [3, 34, 35]. Oral consumption of GA decreased LDH levels (Table 2). GA daily dose had no effect on Erythropoietin level and there was no significant increase in RBCs count. However, the changes in EPO and HbF levels were significantly correlated. Recombinant Erythropoietin alone [36] or in combination with HU [37] is an effective stimulator of fetal hemoglobin production.

This study revealed a novel effect of GA, as oral HbF inducing agent alone or in combination with HU. While HU has a variable therapeutic response [1], in this study 53 % of participants showed response. GA ingestion increase HbF% even in very low baseline HbF% patients. Seven patients with undetectable HbF as measured by electrophoresis, five patients showed elevation of HbF%, absolute change range: (1 · 2–3 · 5). The five respondents were less than 10 years old. The other non-respondents aged 36 and 22. This result showed GA intake may have better response among younger patients.

GA can be utilized as natural safe source of short chain fatty acids by sickle cell anemia patients. In spite of mild effect of GA as fetal inducing agent this effect could be substantial. Patients who had HbF values more than 2 % had a 10-year probability of survival of 89 %, compared with 53 % among patients with HbF lower than 2 % [4].

## Conclusion

In conclusion GA found to increase the percentage of fetal hemoglobin and MCV and decrease MCHC. GA showed no effect on hemoglobin concentration and leucocytes counts.

One of the limitations in our study is not measuring serum butyrate concentration due to resource limitation. Another major limitation in this study we didn’t analyze the effect of GA on gene expression, again because of resource limitation.

This data sheds light on a new era of SCD management that worth further studies. Long duration clinical trials (more than 6 months) and multi arms will be beneficial to assess the sustainability of increase in HbF and its advantages on clinical events and disease severity.

## Additional file

Additional file 1:
**Percentage of HbF of individual subjects.** (PDF 36 kb)

## References

[1] Fathallah H, Atweh GF. Induction of fetal hemoglobin in the treatment of sickle cell disease. Hematology Am Soc Hematol Educ Program 2006;58–62. 10.1182/asheducation-2006.1.58 17124041

[2] Lang E, Lang F (2015). Triggers, inhibitors, mechanisms, and significance of eryptosis: The suicidal erythrocyte death. Biomed Res Int.

[3] Little JA, Hauser KP, Martyr SE, Harris A, Maric I, Morris CR (2009). Hematologic, biochemical, and cardiopulmonary effects of L-arginine supplementation or phosphodiesterase 5 inhibition in patients with sickle cell disease who are on hydroxyurea therapy. Eur J Haematol.

[4] Silva-Pinto AC, Angulo IL, Brunetta DM, Neves FI, Bassi SC, Santis GC (2013). Clinical and hematological effects of hydroxyurea therapy in sickle cell patients: A single-center experience in Brazil. Sao Paulo Med J.

[5] Akinsheye I, Alsultan A, Solovieff N, Ngo D, Baldwin CT, Sebastiani P (2011). Fetal hemoglobin in sickle cell anemia. Blood.

[6] Ware RE, Eggleston B, Redding-Lallinger R, Wang WC, Smith-Whitley K, Daeschner C (2002). Predictors of fetal hemoglobin response in children with sickle cell anemia receiving hydroxyurea therapy. Blood.

[7] Ware RE, Aygun B. Advances in the use of hydroxyurea. Hematology Am Soc Hematol Educ Program 2009;62–9. doi: 10.1182/asheducation-2009.1.62. 10.1182/asheducation-2009.1.62 20008183

[8] Kutlar A, Reid ME, Inati A, Taher AT, Abboud MR, El-Beshlawy A (2013). A dose-escalation phase IIa study of 2,2-dimethylbutyrate (HQK-1001), an oral fetal globin inducer, in sickle cell disease. Am J Hematol.

[9] Diallo DA, Guindo A (2014). Sickle cell disease in sub-Saharan Africa: Stakes and strategies for control of the disease. Curr Opin Hematol.

[10] Atweh GF, Sutton M, Nassif I, Boosalis V, Dover GJ, Wallenstein S (1999). Sustained induction of fetal hemoglobin by pulse butyrate therapy in sickle cell disease. Blood.

[11] Dover GJ, Brusilow S, Samid D (1992). Increased fetal hemoglobin in patients receiving sodium 4-phenylbutyrate. N Engl J Med.

[12] Fathallah H, Weinberg RS, Galperin Y, Sutton M, Atweh GF (2007). Role of epigenetic modifications in normal globin gene regulation and butyrate-mediated induction of fetal hemoglobin. Blood.

[13] Hines P, Dover GJ, Resar LM (2008). Pulsed-dosing with oral sodium phenylbutyrate increases hemoglobin F in a patient with sickle cell anemia. Pediatr Blood Cancer.

[14] Resar LM, Segal JB, Fitzpatric LK, Friedmann A, Brusilow SW, Dover GJ (2002). Induction of fetal hemoglobin synthesis in children with sickle cell anemia on low-dose oral sodium phenylbutyrate therapy. J Pediatr Hematol Oncol.

[15] Ali BH, Ziada A, Blunden G (2009). Biological effects of gum arabic: A review of some recent research. Food Chem Toxicol.

[16] Babiker R, Merghani TH, Elmusharaf K, Badi RM, Lang F, Saeed AM (2012). Effects of Gum Arabic ingestion on body mass index and body fat percentage in healthy adult females: two-arm randomized, placebo controlled, double-blind trial. Nutr J.

[17] Ballal A, Bobbala D, Qadri SM, Foller M, Kempe D, Nasir O (2011). Anti-malarial effect of gum arabic. Malar J.

[18] Tulung B, Remesy C, Demigne C (1987). Specific effect of guar gum or gum arabic on adaptation of cecal digestion to high fiber diets in the rat. J Nutr.

[19] Matsumoto N, Riley S, Fraser D, Al-Assaf S, Ishimura E, Wolever T (2006). Butyrate modulates TGF-beta1 generation and function: Potential renal benefit for Acacia(sen) SUPERGUM (gum arabic)?. Kidney Int.

[20] Nasir O (2013). Renal and extrarenal effects of gum arabic (Acacia senegal)--what can be learned from animal experiments?. Kidney Blood Press Res.

[21] Ali BH, Al-Husseni I, Beegam S, Al-Shukaili A, Nemmar A, Schierling S (2013). Effect of gum arabic on oxidative stress and inflammation in adenine-induced chronic renal failure in rats. PLoS One.

[22] Nasir O, Umbach AT, Rexhepaj R, Ackermann TF, Bhandaru M, Ebrahim A (2012). Effects of gum arabic (Acacia senegal) on renal function in diabetic mice. Kidney Blood Press Res.

[23] Ali BH, Al ZaGÇÖabi M, Ramkumar A, Yasin J, Nemmar A (2014). Anemia in adenine-induced chronic renal failure and the influence of treatment with gum acacia thereon. Physiol Res.

[24] Ali BH, Beegam S, Al Lawati I, Waly MI, Nemmar A (2013). Comparative efficacy of three brands of gum arabic on adenineGÇôinduced chronic renal failure in rats. Physiol Res.

[25] de FL, Corrocher R (2004). Established and experimental treatments for sickle cell disease. Haematologica.

[26] Schechter AN (2008). Hemoglobin research and the origins of molecular medicine. Blood.

[27] Orringer EP, Blythe DS, Johnson AE, Phillips G, Dover GJ, Parker JC (1991). Effects of hydroxyurea on hemoglobin F and water content in the red blood cells of dogs and of patients with sickle cell anemia. Blood.

[28] Ferster A, Tahriri P, Vermylen C, Sturbois G, Corazza F, Fondu P (2001). Five years of experience with hydroxyurea in children and young adults with sickle cell disease. Blood.

[29] Zimmerman SA, Schultz WH, Davis JS, Pickens CV, Mortier NA, Howard TA (2004). Sustained long-term hematologic efficacy of hydroxyurea at maximum tolerated dose in children with sickle cell disease. Blood.

[30] Steinberg MH, Nagel RL, Brugnara C (1997). Cellular effects of hydroxyurea in Hb SC disease. Br J Haematol.

[31] Kinney TR, Helms RW, O’Branski EE, Ohene-Frempong K, Wang W, Daeschner C (1999). Safety of hydroxyurea in children with sickle cell anemia: Results of the HUG-KIDS study, a phase I/II trial. Pediatr Hydroxyurea Group Blood.

[32] Steinberg MH, Voskaridou E, Kutlar A, Loukopoulos D, Koshy M, Ballas SK (2003). Concordant fetal hemoglobin response to hydroxyurea in siblings with sickle cell disease. Am J Hematol.

[33] Fucharoen S, Inati A, Siritanaratku N, Thein SL, Wargin WC, Koussa S (2013). A randomized phase I/II trial of HQK-1001, an oral fetal globin gene inducer, in beta-thalassaemia intermedia and HbE/beta-thalassaemia. Br J Haematol.

[34] Bartolucci P, Brugnara C, Teixeira-Pinto A, Pissard S, Moradkhani K, Jouault H (2012). Erythrocyte density in sickle cell syndromes is associated with specific clinical manifestations and hemolysis. Blood.

[35] Ballas SK, Marcolina MJ (2006). Hyperhemolysis during the evolution of uncomplicated acute painful episodes in patients with sickle cell anemia. Transfusion.

[36] Nagel RL, Vichinsky E, Shah M, Johnson R, Spadacino E, Fabry ME (1993). F reticulocyte response in sickle cell anemia treated with recombinant human erythropoietin: a double-blind study. Blood.

[37] Rodgers GP, Dover GJ, Uyesaka N, Noguchi CT, Schechter AN, Nienhuis AW (1993). Augmentation by erythropoietin of the fetal-hemoglobin response to hydroxyurea in sickle cell disease. N Engl J Med.

