# Supplementary material for: Gum Arabic as fetal hemoglobin inducing agent in sickle cell anemia; in vivo study
Source: BMC Hematol. 2015 Dec 29;15:19. doi: 10.1186/s12878-015-0040-6 (PMC4696138; doi:10.1186/s12878-015-0040-6)
Supplement: Additional file 1: — Percentage of HbF of individual subjects. (PDF 36 kb) [file 12878_2015_40_MOESM1_ESM.pdf]

ID	Medication	Baseline weight	Baseline height	Baseline HbF%	HbF% After 4 weeks	HbF% After 8 weeks	HbF% After 12 weeks
**13P**	FA	21	138	1.8	2	1.8	1.8
**14P**	FA	25	142	2.7	4.6	4.9	2.7
**15P**	FA	42	165	2.7	2.7	3.3	3.5
**16P**	FA	37	159	4.6	5	5.2	4.6
**17P**	FA	15	109	6	6	6.1	7.1
**18P**	FA	48	149	4.5	4.7	5.5	6.3
**20P**	FA	19	130	0	1.9	1.9	1.9
**21P**	FA	17.5	120	0	1.9	1.7	1.7
**22P**	FA	17	124	1.9	1.9	2.2	2
**23P**	FA	15.5	113	13.9	15.2	15.2	15.2
**24P**	FA	22	135	8.9	8.9	8.9	10.6
**25P**	FA	50	167	11.3	11.9	11.9	11.9
**26P**	FA	33	155	4.8	5.4	5.2	5.2
**27P**	FA	32	162	6.4	7.4	5.5	7.4
**28P**	FA	50	170	11	11.8	8.2	11.8
**1ST**	FA	37.5	160	6.8	6.8	5	6.8
**4ST**	FA	45	165	4	4.9	3	4.9
**32P**	FA	56	163	6.8	6.7	6.7	6.7
**33p**	FA	45	190	4.8	5.4	4.1	4.7
**34P**	FA	15	110	2.9	4.4	4.4	4.4
**35P**	FA	18	138	1.9	1.9	1.9	2
**36P**	FA	49	162	9.3	10	10	10
**37P**	FA	35	145	15.5	15.5	15.5	15.5
**38P**	FA	21	130	3.3	3.3	3.3	3.3
**40P**	FA	33	159	13.2	13.2	13.2	13.2
**41p**	FA	13	119	0	2.8	2.8	3.5
**42P**	FA	44	175	0	0	0	0
**44P**	FA	40	157	3.3	3.6	3.6	3.6
**47P**	FA	30	145	0	1.5	1.5	1.6
**48P**	FA	18	120	17.5	17.5	17.5	17.5
**50P**	FA	30	134	0	1.2	1.2	1.2
**51P**	FA	63	180	1.5	2.6	2.6	2.7
**53P**	FA	62	167	16.7	16.8	18	18
**54P**	FA	45	162	2.7	2.9	2.9	2.9
**55P**	FA	23.5	133	1.9	2.1	2.5	2.1
**56P**	FA	14	107	8	10	10	8
**57P**	FA	60	174	5.3	5.7	5.7	6
**58P**	FA	21	126	8.2	10.2	10.3	9
**59P**	FA	43.6	160	0	0	0	0
**61P**	FA	47	143	15.5	16.8		16
**H2**	FA/HU	59	154	11.8	13.7	13.5	11.1

**H3**	FA/HU	63	171	13	13	11	15.7
**H4**	FA/HU	47	162	16	15	15	13.4
**H5**	FA/HU	49	172	12.1	13.5		13.1
**H6**	FA/HU	49	160	15.5	16.8	17.6	16.3
**H7**	FA	44	170	12.2	13.7	15.2	15.2
**H8**	FA/HU	41	150	3.8	5.9	4.7	5.2
